# Estimation of the real population and its impact on the utilisation of healthcare services in Mediterranean resort regions: an ecological study

**DOI:** 10.1186/1472-6963-7-13

**Published:** 2007-01-31

**Authors:** Emilio Perea-Milla, Sergi Mari Pons, Francisco Rivas-Ruiz, Anna Gallofre, Enrique Navarro Jurado, Marco A Navarro Ales, Alberto Jimenez-Puente, Fidel Fernandez-Nieto, Joan C March Cerda, Manuel Carrasco, Lydia Martin, Damian Lopez Cano, Gonzalo E Gutierrez, Rafael Cortes Macías, Jose A Garcia-Ruiz

**Affiliations:** 1 Unidad de Apoyo a la Investigación, Evaluación y Contabilidad (Red IRYSS), Hospital Costa del Sol, Ctra Nacional 340, km 187, 29600 Marbella, Spain; 2 l' Observatori Socioambiental de Menoría, Camí des Castell, 28 1r pis, 07702 Maó, Spain; 3 Departamento de Geografía, Facultad de Filosofía y Letras, Universidad de Málaga, 29071 Málaga, Spain; 4 Mancomunidad Municipios de la Costa del Sol Occidental, Urb. Elviria, N-340, Km. 190, 29600 Marbella, Spain; 5 Delegación provincial de Salud de Málaga, C/Castelao 8, 29004 Málaga, Spain; 6 Escuela Andaluza de Salud Pública, Campus Universitario de Cartuja s/n, 18080 Granada, Spain

## Abstract

**Background:**

The demographic structure has a significant influence on the use of healthcare services, as does the size of the population denominators. Very few studies have been published on methods for estimating the real population such as tourist resorts. The lack of information about these problems means there is a corresponding lack of information about the behaviour of populational denominators (the floating population or tourist load) and the effect of this on the use of healthcare services. The objectives of the study were: a) To determine the Municipal Solid Waste (MSW) ratio, per person per day, among populations of known size; b) to estimate, by means of this ratio, the real population in an area where tourist numbers are very significant; and c) to determine the impact on the utilisation of hospital emergency healthcare services of the registered population, in comparison to the non-resident population, in two areas where tourist numbers are very significant.

**Methods:**

An ecological study design was employed. We analysed the Healthcare Districts of the Costa del Sol and the island of Menorca. Both are Spanish territories in the Mediterranean region.

**Results:**

In the two areas analysed, the correlation coefficient between the MSW ratio and admissions to hospital emergency departments exceeded 0.9, with p < 0.001. On the basis of MSW generation ratios, obtained for a control zone and also measured in neighbouring countries, we estimated the real population. For the summer months, when tourist activity is greatest and demand for emergency healthcare at hospitals is highest, this value was found to be double that of the registered population.

**Conclusion:**

The MSW indicator, which is both ecological and indirect, can be used to estimate the real population in areas where population levels vary significantly during the year. This parameter is of interest in planning and dimensioning the provision of healthcare services.

## Background

The demographic structure has a significant influence on the use of healthcare services, as does the size of the population denominators [1]. However, the vast majority of the studies published on the issue concentrate on factors referring to permanent populations. In areas such as tourist resorts, where large structural changes and annual fluctuations occur, the biggest problem lies in estimating the size of the population, before going on to consider its structure.

In the final decades of the 20th century, the coastal areas of developed countries in temperate climate zones advanced dramatically in both social and economic terms. In 1994, it was estimated that two thirds of the world's population lived within 150 km of the coast, and this value was expected to increase to three quarters by the year 2025 [2]. Beyond all doubt, current levels of economic globalisation have favoured the increased mobility of populations [3].

In Europe, the clearest manifestation of coastal development is to be found along the Mediterranean shores from Spain to Greece, a space in which an authentic urban continuum has been created everywhere physically possible [4]. In addition to concentration in terms of land occupation, tourism also presents a high degree of variability in time, with a marked increase in activity during the summer months; this fact heightens the impact on the environment and tends to produce a model that is economically fragile.

Very few studies have been published on methods for estimating the real population [5]. The earliest studies of the mobility of patients within developed countries highlighted disparities in health-influencing factors, among both the host and the incoming populations, together with variability in the treatment offered to the latter and increasing concern among their respective governments about healthcare costs [6-8]. The lack of information about these problems means there is a corresponding lack of information about the behaviour of populational denominators (the floating population or tourist load) and the effect of this on the use of healthcare services, leading to the seriously inadequate planning of healthcare resources. Indeed, this fact has been reported as impeding the estimation of rates of morbidity-mortality [9-12].

The goals of the present study are: a) To determine the real population in a popular tourist area; and b) to evaluate the impact on the utilisation of hospital emergency services by the permanent, registered population and by that of non-residents, in two popular tourist areas.

## Methods

Design: An ecological study was designed. Location: The geographic areas analysed were the Costa del Sol Healthcare District (CSHD) and the island of Menorca, both of which are part of Spain and in the Mediterranean region. In the years 2001–2002, Menorca had an inter-censal population of 77,036 [13]; public healthcare resources consisted of three primary-attention clinics and one hospital (the Verge de Toro Hospital) in the capital, Mahon. During the inter-censal period of 2001–2002, the CSHD served a population of 271,257 and included a public hospital in the town of Marbella (the Hospital Costa del Sol) and seven primary attention clinics.

Instrumentation: The main variable analysed was the monthly generation of MSW in the two study areas during 2001, these data being supplied by the Association of Municipalities of the Western Costa del Sol (Mancomunidad de Municipios de la Costa del Sol Occidental) and by the Socio-Environmental Observatory (Observatorio Socio-Ambiental) of Menorca [14]. In estimating the Kg. of MSW generated per inhabitant per day in the CSHD, the Control Zone (CZ) was taken as all the inland municipalities in the province of Malaga (thus excluding the coastal municipalities, the city of Malaga and a nearby dormitory town); for these areas, the demographic structure was assumed to be stable, presenting no significant changes throughout the year. In the case of Menorca, the direct estimator used was the variable known as Human Pressure (HP), this variable being applicable thanks to Menorca's island identity, and to the fact that all entries and exits of travellers, by sea or air, are recorded daily. Thus, the total population of the island, including the permanent and the 'floating' elements, is always known. The HP variable enables us to obtain the value of the Kg. of MSW generated per inhabitant per day for every month of the year, rather than having to use a single annual value.

An additional variable included was the maximum accommodation capacity, or the maximum number of beds available, estimated from the total recorded population plus the number of beds in registered accommodation facilities plus the number of second homes and unoccupied residential buildings (corrected by a factor of 10% to allow for speculative housing, this being defined as the flats and houses sold within a year of their purchase) multiplied by the mean number of occupants (3.1) [15].

Statistical Analysis: The ratios calculated were applied to the production of MSW in the two study areas in order to estimate the tourist load and its range; a statistical description was achieved by means of measures of central trend and of dispersion. We estimated the Pearson correlation coefficient (after testing for normality with the Kolmogorov-Smirnov test), the coefficient of determination and the 95% confidence intervals for the estimates of the real population and the use of hospital emergency services during the year in which the study was carried out. For Menorca, these values were calculated for the relation between the MSW ratios and HP. The impact on the demand for healthcare services is described by the relative distribution of patients treated by hospital emergency services and by the patients' mean age, calculated monthly, the patients being differentiated by their place of residence within the reference healthcare district. The healthcare utilisation rate was calculated from the estimated real population (assuming a MSW production of 1.4 Kg. per inhabitant per day). The level of significance was taken as p < 0.05.

## Results

The ratio of Kg. of MSW produced per registered inhabitant per day varied between 2.45 for the CSHD and 1.23 for the CZ. When the real population of the CSHD was estimated, based on three different ratios for the generation of MSW per inhabitant (1.2, 1.4 and 1.6 Kg./person/day), the real population thus estimated exceeded the official values in every case. The estimated monthly population ranged from the 341,000 inhabitants calculated for the month of February, with a ratio of 1.6, to a maximum of 743,000 inhabitants in August, calculated using a MSW ratio of 1.2. The number of estimated inhabitants did not exceed the maximum accommodation capacity except the value calculated for the summer period with a MSW ratio of 1.2. (Table 1) Similar results were obtained for the estimated real population of Menorca using the HP parameter; during May-October, this value was double that of the official figure, while for the remaining months of the year the official value and the HP estimate coincided. The population level was minimum in January, with 70,000 inhabitants, and maximum in August with 177,000. During the months of January to March and in November and December, the estimated real population was less than the official recorded value.

**Table 1:** Estimate of the real population (1000s inhabitants) of the Costa del Sol Health District based on MSW in 2001 and Menorca based on Human Pressure during 2001

	Costa del Sol HD					Menorca		
	
	Registered Population	Max. Accomm. Capacity	Real population estimated with MSW ratio (kg/hab/day)			Registered Population	Max. Accomm. Capacity	Human Pressure
						
			1.2	1.4	1.6			
January	264	631	455	390	341	75	190	70
February	266	633	455	390	341	76	191	71
March	267	634	486	416	364	76	191	72
April	268	635	532	456	399	76	191	83
May	269	636	519	445	389	76	191	118
June	271	638	551	472	413	77	192	141
July	272	639	653	560	490	77	192	160
August	273	640	743	637	557	77	192	177
September	274	641	576	494	432	77	192	141
October	276	643	537	460	403	78	193	107
November	277	644	487	417	365	78	193	73
December	278	645	469	402	352	78	193	71

The correlation coefficient between MSW and HP in Menorca was 0.99 (Table 2). The MSW/HP ratios obtained ranged between 1.38 Kg./person/day in December and 1.55 Kg./person/day in January. In both Menorca and the CSHD, the degree of correlation between the MSW and the monthly number of cases treated at the emergency department of the hospital in the study areas exceeded 0.90 (p < 0.001).

**Table 2:** Bivariate correlation between the generation of Municipal Solid Waste (in tonnes) and Admissions to Emergency Healthcare Hospital Department for Healthcare Districts of the Costa del Sol and Menorca during 2001, and Bivariate correlation between MSW and Human Pressure with MSW ratios for Menorca during 2001

	Costa del Sol HD		Menorca				
	
	MSW	Admissions	MSW	Admissions	MSW	HP	Ratio
January	16935	8094	3377	1714	3377	70088	1,55
February	15275	7756	2915	1523	2915	71297	1,46
March	18069	8534	3515	1837	3515	72096	1,57
April	19141	8859	3969	1777	3969	83065	1,59
May	19312	8919	5439	2019	5439	118345	1,48
June	19845	9047	6128	2179	6128	141212	1,45
July	24308	10190	7097	2400	7097	160269	1,43
August	27628	11652	7726	2686	7726	177344	1,41
September	20743	8206	6450	1981	6450	141383	1,52
October	19982	8293	5274	1894	5274	106975	1,59
November	17520	7479	3470	1532	3470	72591	1,59
December	17437	7722	3030	1644	3030	70911	1,38

C. Correlation	0,93	p < 0,001	0,93	p < 0,001	0,99	p < 0,001	
C.I. 95% lower upper	0,81	0,98	0,82	0,98	0,97	1,00	
C. Determination	0,86		0,87		0,98		

With respect to the impact on health care services, the unregistered population constituted up to 34.9% of all urgent admissions during the month of August in the Costa del Sol Health District, and up to 26.1% in Menorca in the same period. The mean age of non-residents decreased during the summer months in both areas, in comparison with that of the resident population, for whom the corresponding values remained stable (Table 3).

**Table 3:** Relative distribution and mean age of patients and percentage on the total of monthly admissions treated at hospital emergency departments in the Costa del Sol Health District and in Menorca, during 2001

	Costa del Sol HD				Menorca			
	
	Emergency Admission of Unregistered		Mean Age		Emergency Admission of Unregistered		Mean Age	
	
	n	%	Resident	Non- Resident	n	%	Resident	Non- Resident
January	952	11,7	37,6	41,4	41	2,6	41,6	57,0
February	980	12,7	37,0	41,0	42	2,8	39,2	52,6
March	1195	14,0	37,3	41,4	98	4,7	39,3	54,3
April	1674	18,9	36,9	35,6	94	5,7	41,2	34,9
May	1316	14,8	37,3	40,4	120	6,7	40,0	36,5
June	1563	17,3	36,9	36,1	231	9,2	38,3	35,2
July	2757	27,1	38,2	32,0	375	17,5	39,5	33,5
August	4076	34,9	37,9	31,3	787	26,1	38,9	29,3
Septem ber	1578	19,3	38,2	38,0	235	12,8	39,6	38,7
October	1260	15,2	37,5	42,3	122	7,1	39,3	38,2
Novem ber	948	12,7	38,4	41,0	62	3,4	39,7	51,9
Decem ber	965	12,6	37,5	40,7	43	2,8	43,7	49,0

Average	19264	18,4	37,5	36,6	2250	9,7	39,9	35,7

In the year 2001 for the CSHD, hospital emergency service facilities were used by 25.9 per 1000 of the recorded, permanent population, with a difference of 4.3 points between the months of maximum and minimum frequency (Table 4). The permanent population utilised hospital emergency service facilities about 3–4 times more frequently than the non-resident population, this proportion remaining basically constant during the year, although with a slight decrease during the months of July and August, coinciding with the changing ratio of the non-recorded to the official population (exceeding 1.0).

**Table 4:** Population of the Costa del Sol estimated using MSW ratio (1.4 kg./person/day) compared with the registered population and the utilisation of hospital emergency healthcare facilities per 1000 inhabitants per year in both geographic areas during 2001

	Real Population Estimated	Registered Population (RP)	Unregistered Population (URP)	Population URP/RP	Emergency Admissions per 1000 RP	Emergency Admissions per 1000 RP	Admissions URP/RP
January	390.216	264.231	125.985	0,48	26,3	7,3	0,28
February	389.678	265.508	124.170	0,47	27,3	8,5	0,31
March	416.340	266.786	149.554	0,56	26,7	7,7	0,29
April	455.732	268.063	187.669	0,70	26,9	8,9	0,33
May	444.976	269.340	175.636	0,65	27,3	7,3	0,27
June	472.493	270.618	201.875	0,75	27,7	7,7	0,28
July	560.096	271.895	288.201	1,06	26,5	9,3	0,35
August	636.588	273.173	363.415	1,33	27,0	10,9	0,40
September	493.873	274.450	219.423	0,80	24,1	7,2	0,30
October	460.405	275.727	184.678	0,67	24,6	6,6	0,27
November	417.146	277.005	140.141	0,51	23,6	6,8	0,29
December	401.777	278.282	123.495	0,44	23,4	7,6	0,32
Average	462.221	271.257	190.964	0,70	25,9	8,3	0,32

## Discussion

The production of MSW and the population estimates based on this parameter are positively related to the utilisation of emergency healthcare services at hospitals in two geographic areas where tourist activity is significant. The frequency of use of hospital emergency service facilities varies during the year, as does the user profile.

Prior to obtaining the above correlations, we examined indirect indicators that might be used to establish the real population, such as the consumption of electricity, water, cement or hydrocarbon fuels, or the intensity of road traffic, but only the generation of MSW correlated strongly with variations in population and with the use of emergency healthcare facilities.

The choice of standard ratios for the generation of MSW to estimate population levels might introduce a degree of bias, as the areas analysed present different demographic and economic patterns. However, the ratios obtained in the Malaga Control Zone match the ratio calculated under the National Plan for Urban Waste 2000–2006 (1.23 vs. 1.20 Kg./person/day, respectively) [16], while in the coastal area of the same province this ratio was doubled. This phenomenon does not seem to be explained by a corresponding difference in income levels (in the province of Malaga, the weighted mean annual income in the CZ in 2002 exceeded 7,500 euros, while in the CSHD municipalities it exceeded 10,500 euros, only 40% higher). Neither do we observe a systematic increase in the generation of MSW in different countries as income increases [17]. The ranges of ratios applied in this study are close to those measured in European Union countries in 2001, varying between 1.2 Kg./person/day (in Belgium, Portugal and Greece) and 1.6 Kg./person/day (in Germany, the United Kingdom and Holland). The mean value for the 25 countries of the EU is 1.4 Kg./person/day [18].

The estimates of real population based on the generation of MSW reveal a seasonal pattern: in the summer months, the estimated population doubles the official value. In the island of Menorca, seasonality is even greater, as in the first and fourth quarters of the year, the estimated and the officially recorded population values converge, while in the CSHD there exists an unrecorded population that remains stable during the winter months. This latter fact is consistent with the existence of different policies for tourism. On the island of Menorca, the traditional way of life, land and nature have not been displaced by mass tourism, while on the Costa del Sol there is a predominant service industry that is gradually becoming less seasonal by the integration of new areas of leisure activity.

Comparison of estimates of the real population with the utilisation of healthcare resources enables us to evaluate the consistency of these findings, as the greater the population to be attended, the greater the demand for healthcare. Nevertheless, it should be taken into account that the non-resident population normally suffers fewer health problems than the permanent population. The unrecorded population that is resident in winter is usually older, being mainly European pensioners. Among hospital activities, that of the emergency departments presents the highest degree of association with MSW production, as the variability of outpatient treatment and hospital admissions is influenced by non-populational factors that are difficult to control, such as variations in healthcare personnel or changes in programmed activities. On the other hand, either of these populations (registered or unregistered) might seek other sources of care; the use of other health care facilities in primary care or the private sector might be alternative routes to care by these populations.

The correlation coefficients between the number of emergency admissions and MSW production (exceeding 0.90) indicates a strong degree of association and explains the high percentage of variability in the use of emergency healthcare facilities. It should be stressed that the correlation coefficient was identical in the two study areas. This finding is consistent with the association found between MSW production and overnight stays at winter sports resorts in France [5].

The non-resident population of the Costa del Sol is basically made up of three groups: 1) European pensioners, especially from the United Kingdom, Germany and Scandinavia, who stay in the area for several months a year; 2) tourists, both from other areas of Spain and from many foreign countries, who visit the area mainly in summer, but also during other periods, taking advantage of the mild climate; and 3) the population living in neighbouring municipalities who work in the area, mainly in the catering and construction industries. Rates of utilisation of the emergency department at the Hospital Costa del Sol are stable throughout the year, both among the recorded and the unrecorded populations.

The European pensioners make a similar use of healthcare facilities to that of the permanent local population in the same age group, although many of them are not officially registered as resident in Spain. Thus there is an added demand for healthcare and other services (municipal services, infrastructure and communications), the funding for which is provided on the basis of the officially recorded population. The relative increase in the rate of utilisation of healthcare facilities during the months of greatest tourist activity (July and August), together with the fall in the mean age of patients attended, reflect the profile of a younger non-resident population with a higher level of open-air leisure activities, associated with a different morbidity profile. No analysis of healthcare facility utilisation is given for the island of Menorca because the estimation method was influenced by the seasonal economic cycle characterising the island (i.e., the fact that a greater population decrease occurs during the winter), which resulted in negative values being obtained for certain months.

The satisfactory results of the correlation analysis between MSW and HP underline the consistency of MSW as an indirect indicator of the real population, given the reliability of the HP variable in Menorca. This is of great importance, as in the case of the island no physical access is possible except that included in evaluating the HP variable.

## Conclusion

Indicators such as the generation of MSW, which are ecological and indirect, are useful for estimating the total population in areas in which there occur significant changes during the year, if other types of indicators are not available. Annual variations in MSW generation are closely linked to the utilisation of healthcare services, and particularly of emergency hospital services. Such an estimation method is of interest for the planning and dimensioning of the supply of healthcare services, and could be used in other countries in the UE, given the availability of UE MSW data within the EUROSTAT system. Future studies are required to further investigate the structural characterisation of the tourist load and of the needs for healthcare services, broken down by age and sex, among other categories.

## Competing interests

The author(s) declare that they have no competing interests.

## Authors' contributions

All authors contributed to the design of the study. Revision of the different versions of the study protocol: EPM, SMP, FFN, JAGR. Substantial contributions to the conception and design of the digital data record: EPML, FRR, AG. Acquisition of data and quality control: FRR, AG, GEG, MANA, FFN, JCMC. Analysis and interpretation of data: EPM, FRR, MC, LM. All authors have read and approved the final manuscript.

## Pre-publication history

The pre-publication history for this paper can be accessed here:


